# Indications for stent omission after ureteroscopic lithotripsy defined: A single-institution experience with cost analysis

**DOI:** 10.1080/2090598X.2019.1614243

**Published:** 2019-05-16

**Authors:** Paul E. Bower, Jorge Pereira, Osama Al-Alao, Ohad Kott, Danielle Velez, Simone Thavaseelan, Gyan Pareek

**Affiliations:** Section of Minimally Invasive Urology, Alpert Medical School, Brown University, Providence, RI, USA

**Keywords:** Ureteroscopy, stone complications, ureteric stent, stent omission, nephrolithiasis

## Abstract

**Objectives**: To report on our experience with the use of an evidence-based algorithm defining specific indications for stent omission (SO) after ureteroscopic lithotripsy (URSL), as stent placement has been associated with increased cost and morbidity and indications for SO in the setting of uncomplicated ureteroscopy have been proposed but remain vague.

**Patients and methods**: Indications for SO were defined as per the attached figure, data from URSL procedures performed from January 2016 to September 2017 were collected. For procedures eligible for SO, preoperative and intraoperative factors were recorded including: stone burden, presence of preoperative stent, procedure time, access sheath use, and whether SO was performed. Morbidity data were reviewed including: postoperative events, patient telephone calls for bothersome symptoms, unplanned return visits, and admissions within 30 days.

**Results**: In all, 250 URSL procedures were performed during the study period, and 106 (42.4%) were eligible for SO. SO was performed in 60 (24.0%) cases reflecting a 56.7% compliance with the algorithm. There were no readmissions or re-operations within 30 days for the SO group. Lower postoperative event rates were noted in the SO group (16.7% vs 34.8%, *P* = 0.03), unplanned return visits (8.3% vs 17.4%, *P* = 0.16) and 30-day readmission rates (0.0% vs 6.5%, *P* = 0.08) were also lower in the SO group, although they did not reach statistical significance. Analysis also demonstrated a protective effect of SO on unplanned return visits (odds ratio 0.43, 95% confidence interval 0.13–1.42, *P* = 0.17), although this was not statistically significant. No statistically significant associations were noted between postoperative events and stone burden, procedure time, or presence of preoperative stent.

**Conclusions**: We provide an algorithm defining indications for SO. SO is safe in a significant portion of URSL procedures, and SO appears to decrease postoperative events when performed judiciously.

**Abbreviations:** IQR: interquartile range; LUTS: lower urinary tract symptoms; OR, odds ratio; SO: stent omission; URSL: ureteroscopic lithotripsy; YAG: yttrium-aluminium-garnet

## Introduction

Ureteric stents have been associated with significant postoperative morbidity [1] and increased cost when placed unnecessarily [2]. Although AUA guidelines state stents may be omitted for uncomplicated ureteroscopic lithotripsy (URSL), specific indications to guide urologists remain elusive [3]. Randomised controlled trials have shown that stent omission (SO) yields decreased postoperative pain and symptoms in uncomplicated URSL, but these reports have not translated into lower rates of stent placement in the USA. Data has been largely limited to ureteric stones without the use of ureteric access sheaths [4–7], and a recent meta-analysis showed a possible increase in unplanned postoperative visits for patients where SO was performed [8]. Thus, although stent use varies widely internationally, it remains nearly ubiquitous in the USA, with stents being placed after 93% of URSL procedures [9]. In comparison, the UK places stents after 70% of URSL procedures. That difference may be driven by fear of unplanned follow-up visits.

The development of a clear definition for uncomplicated ureteroscopy is needed to show where a stent could be safely omitted. Our objective in the present study was to establish judicious and specific indications for SO based on a retrospective analysis of outcomes and cost benefit.

## Patients and methods

After review of the available literature and AUA guidelines indications for SO after URSL were defined as patients meeting all the following criteria and either criterion A or criterion B:

Normal contralateral kidney, normal renal function, no evidence of ureteric stricture or other anatomical impediments to stone fragment clearance, no suspected ureteric injury during the procedure, no secondary ureteroscopic procedure planned and either A) Patients were pre-stented, or B) Renal or distal ureteric stone without access sheath placement during the procedure.

Following Institutional Review Board approval, cost analysis of SO was evaluated in a cohort of 126 patients that underwent URSL between January and September 2016. The cost of the stent removal, as well as the cost of postoperative events, were calculated using the Medicare Physician Fee Schedule Look-Up Tool [10]. The cost related to stent insertion was calculated to be $90 (American dollars) based on our direct cost of purchase.

**Figure 1.**
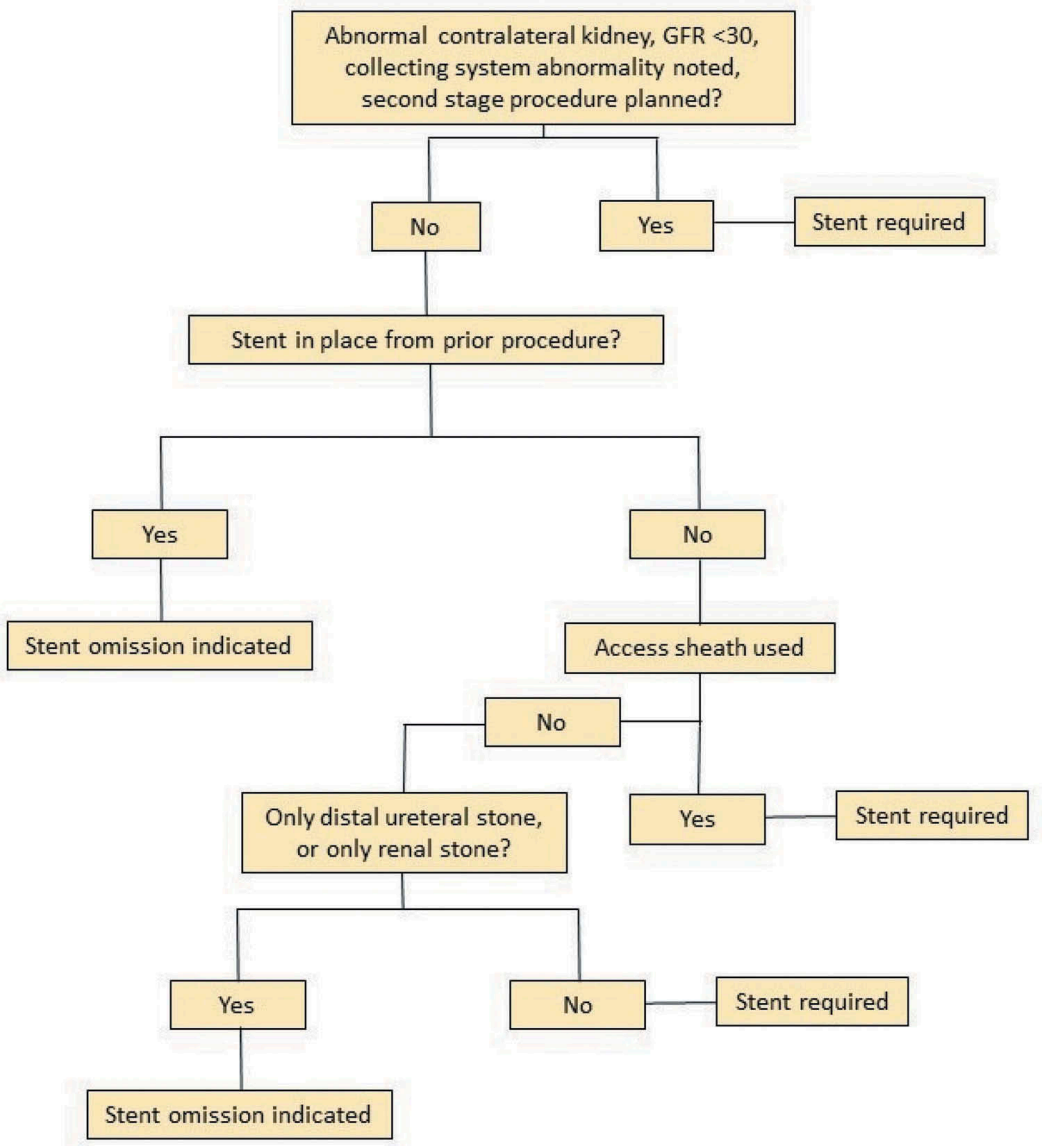
Protocol for SO.

After analysis of this cohort and review of available evidence; a protocol for SO was created as shown in Figure 1. According to the protocol, SO is indicated in patients that were either pre-stented or that did not require access sheath placement. A second cohort was used to evaluate the morbidity of SO using the aforementioned protocol and included 280 patients that underwent URSL between November 2016 and September 2017. In procedures eligible for SO, preoperative and intraoperative factors were recorded including: stone burden, presence of preoperative stent, procedure time, access sheath use, and whether SO was performed or not. Postoperative events were recorded including: any telephone call for symptoms, unplanned office visits, emergency department visits or admissions within 30 days of the procedure.

Patients who were pregnant, those with pre-existing nephrostomy access at the time of the procedure, those undergoing bilateral procedures, and those undergoing simultaneous bladder outlet procedures were excluded from the analysis. Ureteroscopy was performed with either a semi-rigid (Karl Storz 27010K 6 Degree, 7-F; Karl Storz GmbH & Co. KG, Tuttlingen, Germany) or flexible ureteroscope (Flex X2, Karl Storz). Ureteric access sheaths (Cook Flexor, Cook Urological, Bloomington, IN, USA) used were 20–35 cm for women and 35–45 cm for men and with 12/14 F diameter. Stones were fragmented using a holmium:yttrium-aluminium-garnet (YAG) laser, and stone fragments >2 mm were extracted with a basket under direct vision. After lithotripsy and extraction, the collecting system was examined for injury. A 6-F Inlay stent (Bard Urological, Covington, GA, USA) was used when necessary. Balloon dilatation was not performed during the study period in any URSL procedure.

Patients’ baseline characteristics, perioperative variables, and outcomes were summarised using medians/interquartile ranges (IQRs) and frequency counts/percentages, and compared using ANOVA and Pearson chi-squared test or Fisher’s exact test, respectively. Univariate logistic regression was used to evaluate the associations of patient or operative features with the occurrence of a postoperative event. Effect estimates were summarised using odds ratios (ORs) with 95% CIs. Statistical analyses were performed using Stata version 14.2 (StataCorp, College Station, TX, USA). All tests were two-sided, and *P* values < 0.05 were considered to be statistically significant.

## Results

### Cost analysis

A total of 126 URSLs were identified from January to September 2016, and 67 (53.2%) patients met the criteria of SO. Out of those, SO was performed in nine patients and 58 patients had a stent placed. Cost analysis was performed on those 58 patients were SO was indicated but not done (Table 1).

**Table 1.** Stent placement and SO rates before SO protocol implementation.

Variable, *n* (%)	Met criteria for SO	Did not meet criteria for SO	Total, *n* (%)
Total stents placed	58 (86.6)	59	117 (92.9)
With postoperative events	23 (39.7)		
Without postoperative events	35 (60.3)		
Stent omitted	9 (13.4)	0	9 (7.1)
With postoperative events	0		
Without postoperative events	9/9		
Total	67 (100)	59	126 (100)

Postoperative events occurred in 23 of the 58 patients. No events occurred in the nine patients where SO was indicated and performed. The difference in postoperative events rate between these two groups was statistically significant (*P* = 0.025). Postoperative events included new flank pain, LUTS, significant haematuria, proximal stent migration, distal stent migration, and need for anaesthesia to remove stent cystoscopically. The cost of unnecessary stent placement was calculated to be $372.45 (American dollars) per patient, with the average cost being $540.09 per patient for those with complications and $272.92 for those patients without complications (Table 2). We calculated our potential cost savings to be $29 424 annually per endourologist. In all, 23 patients who met criteria for SO and were stented had the stent removed via a string, and the average unnecessary cost for these patients was $230.19.

**Table 2.** Potential cost savings in stented cases that met criteria for SO.

Variable	*N* (%)	Potential cost savings per case, $	Total annual potential cost savings per endourologist, $
With postoperative events	23 (39.7)	540.09	16 681
Without postoperative events	35 (60.3)	272.92	12 473
Total	58 (100)	372.45	29 424

$, American dollars.

### Morbidity of SO

**Figure 2.**
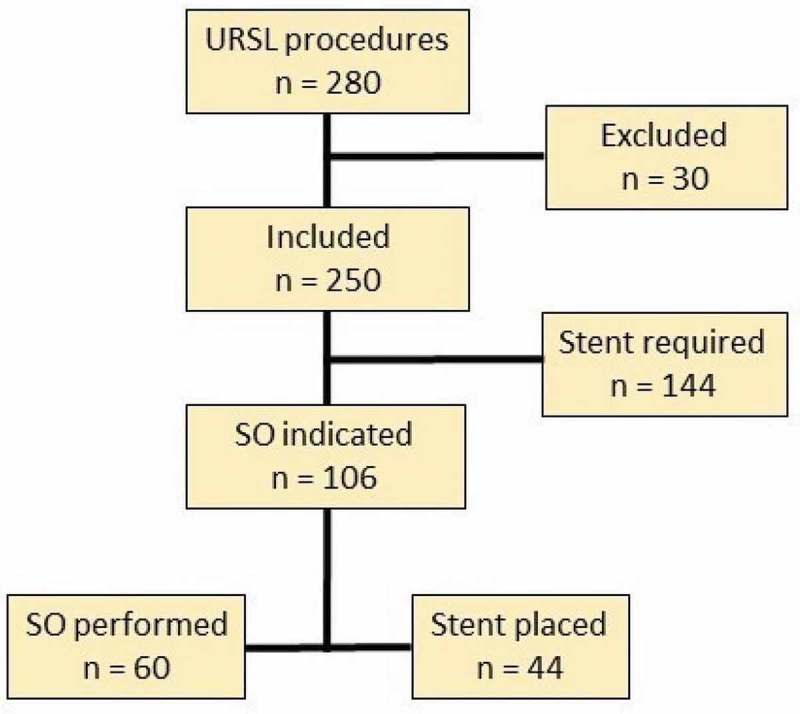
Flowchart of URSL procedures performed from November 2016 to September 2017.

A total of 280 URSL procedures were performed from November 2016 to September 2017 after discussion of the above cost and morbidity analysis (Figure 2). In all, 30 patients were excluded based on the aforementioned criteria. Of the 250 URSL procedures, 72 cases had a ureteric stone (40 lower and 32 upper) and 178 had a kidney stone, and of them 22 cases had kidney and ureteric stones simultaneously. A semi-rigid ureteroscope was used for the 40 cases of distal ureteric stones, and a flexible scope with or without sheath was used for the rest of the cases.

In all, 106 patients (42.4%) were eligible for SO. SO was performed in 60 (24.0%) patients reflecting a 56.7% compliance with the algorithm (Figure 1). Patients where SO was performed were more likely to have shorter median procedure time (18.0 vs 30.0 min, *P* < 0.001) and smaller median stone burden (6.0 vs 9.0 mm, *P* < 0.001), as shown in Table 3.

**Table 3.** Characteristics of patients in which SO was indicated, stratified by performance of SO.

Variable	SO performed (*n* = 60)	Stent placed (*n* = 46)	*P*
Age, years, median (IQR)	55.5 (43.5–63.5)	56 (46–64)	0.55^a^
Female, *n* (%)	29 (45.7)	21 (48.3)	0.78^b^
Stone burden, mm, median (IQR)	6.0 (4.0–8.0)	9.0 (6.0–11.0)	<0.001^a^
Procedure time, min, median (IQR)	18.0 (12.5–26.0)	30.0 (19.0–43.0)	<0.001^a^
Pre-stented, *n* (%)	29 (48.3)	25 (55.5)	0.40^b^
Postoperative event, *n* (%)	10 (16.7)	16 (34.8)	0.03^b^
Unplanned return, *n* (%)	5 (8.3)	8 (17.4)	0.16^b^
Readmission, *n* (%)	0 (0.0)	3 (6.5)	0.08^c^

^a^Kruskal–Wallis; ^b^chi-squared; ^c^Fisher’s exact.

The overall postoperative event rate was 24.8%, and events included fever, sepsis, nausea, flank pain, haematuria, urinary retention, fatigue, LUTS, a fall, and an emergency department visit for discomfort due to a stent string. Lower postoperative event rates were noted in the SO group (16.7% vs 34.8%, *P* = 0.03), unplanned return visits (8.3% vs 17.4%, *P* = 0.16) and 30-day readmission rates (0.0% vs 6.5%, *P* = 0.08) were also lower in the SO group, although this did not reach statistical significance.

Univariate associations with postoperative events are summarised in Table 4. SO was shown to be protective, with patients undergoing SO found to have 0.38-times the odds of experiencing a postoperative event when compared to those who were stented (95% CI 0.15–0.93, *P* = 0.04). No statistically significant associations were noted between postoperative events and stone burden, procedure time, or presence of preoperative stent. There were no readmissions or re-operations within 30 days for the 60 patients in the SO group. Analysis also demonstrated a protective effect of SO on unplanned return visits (OR 0.43, 95% CI 0.13–1.42, *P* = 0.17), although this was not statistically significant.

**Table 4.** Patient characteristics, stratified by postoperative event and univariate association of these characteristics with postoperative event.

	Postoperative events				
Variable	No (*n* = 80)	Yes (*n* = 26)	*P*	OR (95% CI)	*P*
Age, years, median (IQR)	56 (44–64)	55.5 (45–63)	1.00^a^	1.00 (0.97–1.04)	0.97
Female, *n* (%)	37 (46.3)	13 (50.0)	0.79^b^	1.16 (0.48–2.82)	0.74
Stone burden, mm, median (IQR)	7.0 (5.0,10.0)	8.0 (6.0,10.0)	0.12^a^	1.07 (0.95–1.20)	0.25
Procedure time, min, median (IQR)	20.0 (14.0, 30.0)	24.0 (18.0,32.0)	0.16^a^	1.02 (0.99–1.05)	0.26
Pre-stented, *n* (%)	42 (52.5)	13 (50.0)	0.83^b^	0.90 (0.37–2.19)	0.83
SO, *n* (%)	50 (62.5)	10 (38.5)	0.03^b^	0.38 (0.15–0.93)	0.04

^a^Kruskal–Wallis; ^b^chi-squared.

An access sheath was used on 24 patients who were eligible for SO, and a stent was omitted in six patients. Of those six patients, one patient had a postoperative event of urinary retention discovered on the first postoperative day, and he passed trial of void on postoperative day 5. SO was not significantly associated with postoperative events for this group of patients (OR 0.52, 95% CI 0.05–5.63, *P* = 0.59).

## Discussion

The present study defines indications for SO based on available evidence, and reports on the outcomes of patient cohort in which these indications were used. We performed a total of 60 consecutive SOs without a readmission or re-operation using the indications for SO shown in Figure 1. We report improvement in morbidity when a stent is omitted compared to when it is placed unnecessarily. These results are consistent with previous studies. Our present study differs from previous studies, as it includes analysis of both renal and ureteric stones, with and without the use of an access sheath. We find 42% of cases in our practice meet criteria for SO. These findings reaffirm the current AUA Guidelines that SO may be performed after uncomplicated ureteroscopy, as well as providing further guidance as to specific circumstances where it is beneficial to omit a stent. It should be noted that SO need only be non-inferior to stent placement to warrant consideration due to cost, although a significant amount of data show that SO improves patient outcomes when performed judiciously [4,5,7,11].

Stents were omitted in 24% of cases after SO indications were prospectively utilised, reflecting a 240% increase in SO and a 56.7% compliance with the algorithm provided. Reasons for lack of compliance include cases where a second-look was needed or due to intraoperative findings, such as stones impaction and ureteric oedema. Although patients who were eligible for SO and received a stent had greater stone burden and longer procedure times, we found postoperative events not to be associated with procedure time or stone burden. A multivariate analysis of factors associated with postoperative events could be considered given the preoperative differences in the stented and SO groups. However, only SO had a *P* < 0.2, which obviated the need for a multivariate analysis.

Denstedt et al. [4] showed in 2001 that routine stent placement was not uniformly necessary in the setting of URSL of ureteric stones with a holmium:YAG laser and basketting. Results showed an improvement in pain scores at 1 week, but otherwise equivalent results for stented and unstented patients. Excluded from this study were patients who had balloon dilatation and pre-stented patients. In addition, the data were not stratified into distal or proximal ureteric stones. Borboroglu et al. [6] showed a clear improvement in pain when stents were omitted for patients with distal stones undergoing URSL. Although pain scores were significantly better in the unstented group, all four readmissions were in the unstented group. Semi-rigid ureteroscopes as large as 9-F were used, and patients were included whether or not they had dilatation performed. Subsequent randomised controlled trials have also shown SO to improve postoperative pain for distal stones or procedures where only semi-rigid ureteroscopes were used [5,7]. This led to our inclusion of distal stones as eligible for SO. However, we assigned all patients with a proximal stone who were not pre-stented to have a stent placed, as there is not enough evidence on SO for proximal stones. Indications for SO may be expanded in the future to include this subset of patients after further study. Patients who are not pre-stented and who have both ureteric and renal stones may also eventually be found to not require a stent.

Data concerning the use of ureteric access sheath use have not demonstrated conclusive results. In a retrospective review, Rapoport et al. [12] showed a significant increase in Emergency Room visits for patients in whom a ureteric access sheath was used and who had SO. A case-control study by Torricelli et al. [13] also found lower pain scores and follow-up visits for pain for patients where a stent was placed after use of a ureteric access sheath. However, in a subgroup analysis they found pre-stented patients may not require a stent. Although small, our present experience agrees with this finding. Of the six pre-stented patients where an access sheath was used, the only postoperative event was for urinary retention, which is not likely to be caused by SO. Based on our experience and available evidence, the vast majority of pre-stented patients should have a stent omitted, and this remains a key target for compliance in our practice going forward.

Our present data suggests that SO may be protective against unplanned follow-up visits, contrary to the recent meta-analysis performed by Pais et al. [8]. This is not surprising given their inclusion of large trials where our algorithm would suggest a stent be placed, such as in the setting of balloon dilatation of the ureteric orifice [14] or use of an access sheath in a patient who is not pre-stented [12]. With judicious indications for SO established, the possibility exists to review the available data with a meta-analysis that incorporates these indications.

Limitations of the present study include its retrospective design, its reliance on operative findings as previously mentioned, and its lack of a true control group with significant differences between patients where a stent was placed and where a stent was omitted. However, this reflects the real world application of plan-do-study-act to the clinical management of stent practices.

## Conclusions

We provide indications for SO and report on our experience of 60 consecutive cases of SO without readmission or re-operation. Stents are clearly overused in the USA at this time and given the cost associated with their unnecessary use, as well as patient discomfort, optimising SO addresses both patient and cost outcomes. Although SO appears to improve morbidity and could potentially reduce unplanned follow-up visits when performed according to these indications, prospective data are needed.
